# Automatic quantification of superficial foveal avascular zone in optical coherence tomography angiography implemented with deep learning

**DOI:** 10.1186/s42492-019-0031-8

**Published:** 2019-12-09

**Authors:** Menglin Guo, Mei Zhao, Allen M. Y. Cheong, Houjiao Dai, Andrew K. C. Lam, Yongjin Zhou

**Affiliations:** 1 School of Biomedical Engineering, Health Science Center, Shenzhen University, Shenzhen University Xili Campus, Room 208, Block A2,, Taoyuan Street, Shenzhen, 518055 China; 2 Centre for Myopia Research, School of Optometry, Faculty of Health and Social Sciences, The Hong Kong Polytechnic University, Kowloon, Hong Kong, China

**Keywords:** Optical coherence tomography angiography, Deep learning, Foveal avascular zone, Automatic segmentation and quantification

## Abstract

An accurate segmentation and quantification of the superficial foveal avascular zone (sFAZ) is important to facilitate the diagnosis and treatment of many retinal diseases, such as diabetic retinopathy and retinal vein occlusion. We proposed a method based on deep learning for the automatic segmentation and quantification of the sFAZ in optical coherence tomography angiography (OCTA) images with robustness to brightness and contrast (B/C) variations. A dataset of 405 OCTA images from 45 participants was acquired with Zeiss Cirrus HD-OCT 5000 and the ground truth (GT) was manually segmented subsequently. A deep learning network with an encoder–decoder architecture was created to classify each pixel into an sFAZ or non-sFAZ class. Subsequently, we applied largest-connected-region extraction and hole-filling to fine-tune the automatic segmentation results. A maximum mean dice similarity coefficient (DSC) of 0.976 ± 0.011 was obtained when the automatic segmentation results were compared against the GT. The correlation coefficient between the area calculated from the automatic segmentation results and that calculated from the GT was 0.997. In all nine parameter groups with various brightness/contrast, all the DSCs of the proposed method were higher than 0.96. The proposed method achieved better performance in the sFAZ segmentation and quantification compared to two previously reported methods. In conclusion, we proposed and successfully verified an automatic sFAZ segmentation and quantification method based on deep learning with robustness to B/C variations. For clinical applications, this is an important progress in creating an automated segmentation and quantification applicable to clinical analysis.

## Introduction

Optical coherence tomography (OCT) has significantly advanced ophthalmic imaging, and OCT angiography (OCTA) is a noninvasive approach that provides a high-resolution visualization of the vasculature in the retina and choroid without the injection of an intravenous contrast [1]. With the advent of high-speed OCT and efficient algorithms, OCTA has been used to evaluate diabetic retinopathy [2], retinal vein occlusion [3], nonexudative age-related macular degeneration [4], and macular telangiectasia type 2 [5].

Classical histology publications expound that two parallel vascular networks exist at the inner retinal level [6], the superficial network and the deep network. The foveal avascular zone (FAZ), a region of the fovea without blood, consists of a superficial FAZ (sFAZ) at the superficial level of the retina and the deep FAZ (dFAZ) at the deep level of the retina. The areas of the sFAZ of both patients with diabetic retinopathy and patients with retinal vein occlusion are larger than those of healthy people [7, 8]. Meanwhile, the sFAZ is negatively correlated with the best-corrected visual acuity [9]. In all eyes, the sFAZ is positively correlated with the logarithm of the minimum angle of resolution of visual acuity [10]. Hence, accurate segmentation and quantification of sFAZ is crucial for the diagnosis and treatment of the abovementioned diseases.

Several OCTA devices provide images with different brightness and contrast (B/C) variations. A method to obtain quantitative metrics of vascular plexuses is to export OCTA images and use publicly available image processing software such as ImageJ to manually analyze the images. The process used by ophthalmologists, i.e., manually analyzing vascular plexuses to segment the sFAZ, is labor intensive and time consuming. Meanwhile, the analysis results may vary according to the B/C settings [11]. Binarization thresholding and B/C adjustments can significantly affect quantitative metrics. Compared to manual segmentation, automatic segmentation can be more efficient, reliable, and objective. Unfortunately, automatic segmentation methods previously reported, which are based on human prior knowledge, exhibit the following problems:

1. Although these methods may perform well on singular OCTA datasets, they cannot accurately segment the sFAZ in the OCTA images from other datasets by overfitting to the original sample.
2. The parameters of these methods are selected empirically, thereby causing the automatic segmentation results of the sFAZ to be destroyed significantly when the B/C of the image are different from the default.

Specifically, Lu et al. [12] extracted an sFAZ by applying a region-growing approach, in which the image center point was manually selected as a seed. The final sFAZ segmentation result was obtained by applying morphological operators and an active contour model. Díaz et al. [13] identified all potential FAZ (both sFAZ and dFAZ) candidates by applying morphological operators and edge detection techniques. Subsequently, specific domain knowledge was used to preserve the most suitable FAZ localization from all the FAZ candidates. Finally, a region-growing approach was applied to the most suitable FAZ localization to obtain the final FAZ segmentation results.

Deep learning is a technology that contributes to the excellent performance for discovering the intricate structures of high-dimensional data. The layer used to extract features in deep learning are learned from data using a general-purpose learning procedure, without relying on the design of human engineers [14]. This allows deep learning to provide highly accurate and objective results and perform even better in generalization with new datasets [14]. Currently, deep learning for the automatic classification and segmentation of OCT images in ophthalmology affords excellent results [15, 16]. Furthermore, deep learning is applicable to image B/C adjustments [17, 18]. Therefore, we herein propose a method based on deep learning for the segmentation and quantification of the sFAZ in OCTA images. The method was designed to be insensitive to variations in B/C, and applied to segment and quantify the sFAZ.

The main purpose of this study is to demonstrate the high accuracy and significant correlation of the proposed method based on deep learning for the segmentation and quantification of the sFAZ in OCTA images with robustness to B/C variations. The low contrast of the dFAZ boundary caused by the dense and complex deep retinal capillary network challenges the accurate segmentation and quantification of the dFAZ. The robustness of the proposed method to B/C variations demonstrates the potential for the accurate segmentation and quantification of the dFAZ.

## Methods

**Fig. 1:**
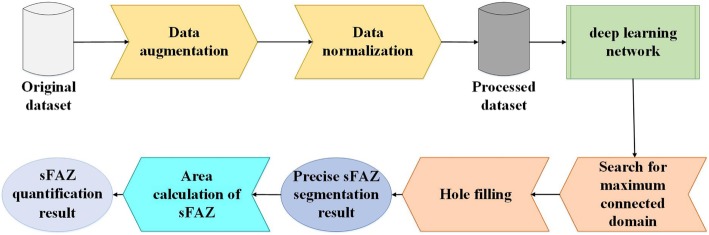
The proposed method pipeline

The pipeline of the proposed method is illustrated in Fig. 1. To accelerate the training process, the original OCTA image data were augmented [19] and normalized [20]. Next, the sFAZ was segmented using our proposed deep learning network. Subsequently, we applied the largest-connected-region extraction and hole-filling to obtain a precise sFAZ segmentation. Finally, the sFAZ quantification results were obtained, as detailed in Section 2.6.

### Image dataset and preparation for training and testing

**Fig. 2:**
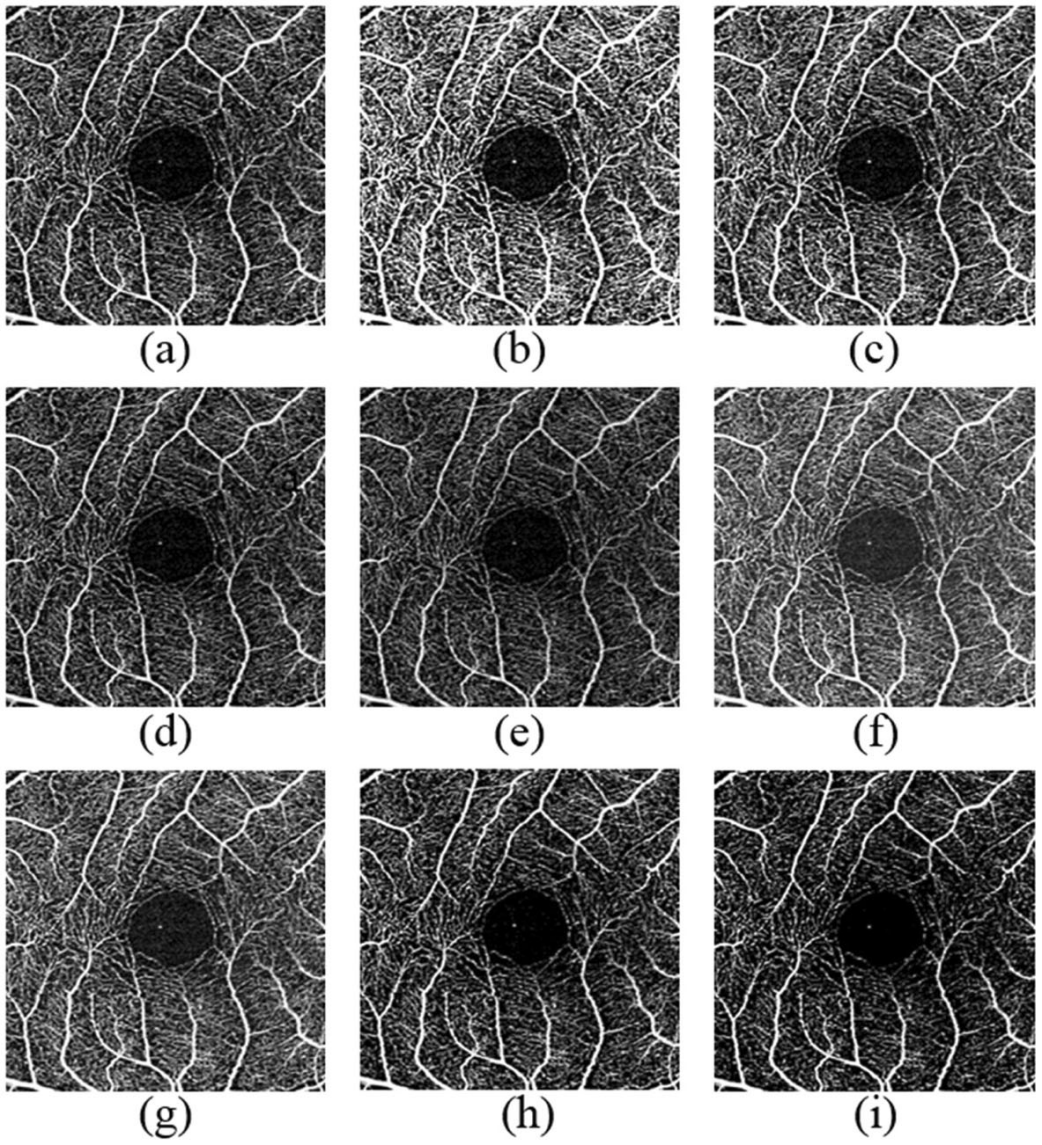
An example of OCTA images with nine different brightness/contrast settings. **a** 130/20; **b** 90/20; **c** 110/20; **d** 150/20; **e** 170/20; **f** 130/0; **g** 130/10; **h** 130/30; **i** 130/40

From April 2017 to August 2018, randomly selected OCTA images of 45 eyes from 45 participants were acquired in the Optometry Clinic of the Hong Kong Polytechnic University, which include those of 22 males and 23 females aged between 18 to 49 years. The tenets of the Declaration of Helsinki were adhered to for the study. Ethics clearance was obtained from the Institution Review Board of the Hong Kong Polytechnic University. Written consent forms were obtained from all participants. Twelve participants were high myopes with a spherical equivalent ≤ −6D, and 33 were low myopes (spherical equivalent > −6D). For each eye, a 3 mm × 3 mm OCTA image centered on the macula was captured using Cirrus HD-OCT 5000 with AngioPlex (Carl Zeiss Meditec, Inc., Dublin, California). Cirrus has an A-scan rate of 68,000 scans per second and a light source with a central wavelength of 840 nm. The axial and transverse resolutions in tissue are 5 and 15 μm/pixel, respectively. The default B/C setting of the superficial retina layer is 130/20 (Group 1). The B/C was manually changed to the following settings: 90/20 (Group 2), 110/20 (Group 3), 150/20 (Group 4), 170/20 (Group 5), 130/0 (Group 6), 130/10 (Group 7), 130/30 (Group 8), and 130/40 (Group 9). An example of OCTA images with nine different brightness/contrast settings is shown in Fig. 2. The default contrast and brightness settings are 20 and 130, respectively. The higher the values (of both contrast and brightness), the darker the images. More capillaries are visible at the contrast from 20 to 10 or the brightness from 130 to 120; however, noise increases. On the contrary, changing the contrast and brightness to higher values resulted in darker images with fewer visible capillaries. At different scales, the capillaries and noise had different degrees of visualization. In total, 405 OCTA images of the superficial retina were exported for image analysis.

The ground truth (GT) of the sFAZ was generated on both training and testing sets by filling the inner area of the sFAZ boundary manually using the “fill” and “polygon selections” programs in the ImageJ software (National Institutes of Health, Bethesda, MD).

### Data preprocessing

A total of 405 OCTA images from 45 participants (12 high myopic and 33 low myopic) were rotated and flipped to 2430 to fulfill the requirement of a large amount of data for training a deep learning network. To accommodate the proposed deep learning network, all OCT images were downsized to a standard resolution of 704 × 704 pixels. To substantially improve the final generalization error of the proposed deep learning network and accelerate training, the original data were normalized by applying Eq. (1):

1 $$ \hat{\mathrm{X}}=\frac{\mathrm{X}-{\mathrm{X}}_{\mathrm{mix}}}{{\mathrm{X}}_{\mathrm{max}}-{\mathrm{X}}_{\mathrm{min}}} $$

$\hat{\mathrm{X}}$ denotes the result of normalization, X the image, X_max_ the maximum pixel value in the image, and X_min_ the minimum pixel value in the image.

### Description of the proposed deep learning network

**Fig. 3:**
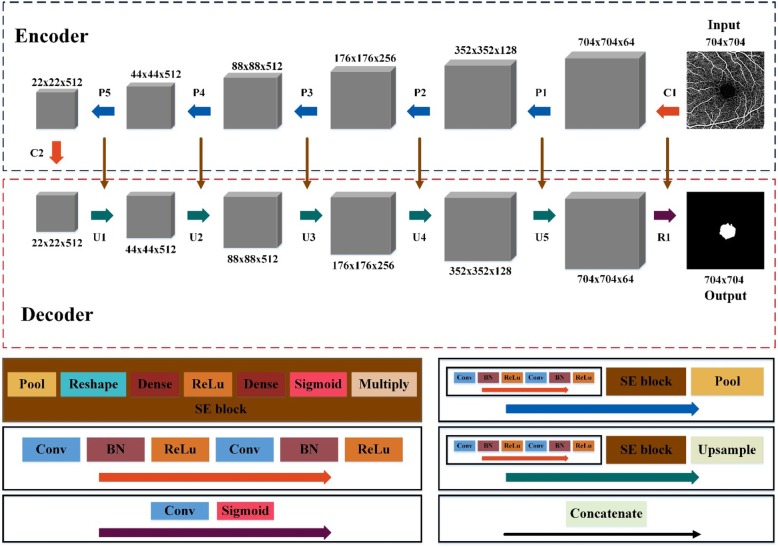
Graphical representation of the proposed deep learning network. The proposed deep learning network included an encoder and decoder. The encoder comprised two Conv-BN- ReLu blocks (C1–C2) and five pooling blocks (P1–P5). The decoder comprised five upsampling blocks (U1–U5) and a reconstruction block (R1). The output of each layer is a three-dimensional feature map of size (h × w × d), where h and w are the height and width of the feature map, respectively, and d is the feature dimension

We designed a deep learning network, inspired by a fully convolutional networ k[21] and U-Net [22], to perform classification among the sFAZ and other regions for each pixel in the OCTA image. An overview is presented in Fig. 3. The proposed network allowed an input image of a specific size to be mapped to an image of corresponding class labels of the same size by automatically extracting the semantic information of the input image. The proposed network consisted of two processing components: an encoder that extracted abstract semantic information from the input image and a decoder that mapped the abstract semantic information to an image of corresponding class labels at the pixel level.

The encoder (C1–C2, P1–P5) included two Conv-Batch normalization (BN )[23] -ReLu blocks (C1–C2) and five pooling blocks (P1–P5). A pooling block included a Conv-BN-ReLu block, a squeeze-and-excitation (SE) block [24], and a pooling layer (Pool); meanwhile, a Conv-BN-ReLu block comprised two convolutional layers (Conv), two BN layers, and two ReLu layers (ReLu). The SE block included one pooling layer, one reshape layer (Reshape), two dense layers (Dense), one ReLu layer, one sigmoid layer (Sigmoid), and one multiply layer (Multiply). The convolutional layer of a sequence of square filters generated a sequence of feature maps containing the semantic information as a result of a two-dimensional convolution. The BN layer performed standardization on each feature map to accelerate the training process of the proposed network and improve the segmentation performance. The activation function ReLu, which improves the nonlinearity of the proposed network, was applied to calculate the output of each feature map. In the SE block, the pooling layer and the reshape layer changed the size of the feature map; subsequently, a parameter between 0 and 1 for each feature map as an input to the SE-block was generated through two dense layers: one ReLu layer and one sigmoid layer. The Multiply layer multiplied each feature map by the corresponding parameter as the output of the SE block. The SE block improved the performance of the proposed network at minimal additional computational costs. The pooling layer reduced the size of the feature map to increase the receptive field by applying the maximum activation over nonoverlapping square regions.

The decoder (U1-U5, R1) reconstructed the sFAZ segmentation result from 22 × 22 pixels to 704 × 704 pixels by applying five upsampling blocks, and a reconstruction block. The upsampling block comprised a Conv-BN-ReLu block, SE block, and upsampling layer (Upsample). A reconstruction block comprised one convolutional layer and one sigmoid layer. The upsampling layer restored the size of the feature maps through bilinear interpolation. The concatenate layer (Concatenate) was designed to solve the problem of potential missing image details in this reconstruction by fusing the feature maps. The feature maps were reconstructed to an image of corresponding class labels of the same size as the original image after finalizing all the upsampling blocks and the reconstruction block.

### Network training strategies

The parameters for the proposed network training process were set as follows:

1. The basic learning rate was reduced to half the basic learning rate of the previous epoch if the loss within 30 epochs did not reduce from an initial value of 1 × 10^− 4^.
2. Ada m[25] was applied as an optimization to guarantee an efficient calculation and robustness to the data noise of the proposed network.
3. The program automatically saved the proposed network model once whenever the correct rate of the testing set had increased.

Based on the Keras framework [26], we used a GPU NVIDIA GeForce GTX 1080TI equipped on an Intel Xeon E5–2650 2.30 GHz machine with a Linux Ubuntu 14.04 operating system to train the proposed network.

### Result post-processing

Some sFAZ segmentation results from the proposed network output included tiny holes and spots. We used the largest-connected-region extraction and hole-filling to provide precise sFAZ segmentation results for all the OCTA images.

### Final sFAZ area calculation

The area of the sFAZ from the segmentation results was calculated using Eq. (2):

2 $$ \mathrm{Area}=\mathrm{N}\times \frac{{\mathrm{mm}}^2}{\mathrm{H}\times \mathrm{W}} $$

N represents the number of pixels of the precise sFAZ segmentation results, mm represents the size in millimeters of the OCTA image, and H and W represent the height and width of the analyzed OCTA image, respectively.

### Cross-validation methods

We tested the proposed method using a five-fold stratified cross-validation on our dataset of 45 participants. All participants, including all nine groups of images for each participant, were randomly divided into five groups (participant based). At every fold, one group was used as the test set without repetition and the remaining were used as the training sets.

### Performance evaluation

When the automatic segmentation result was compared against the GT, each pixel of the automatic segmentation result was classified as either a true positive (TP), true negative (TN), false positive (FP), or false negative (FN). Metrics of dice similarity coefficient (DSC), sensitivity, and specificity were calculated to evaluate the segmentation performance of the proposed method. The equation of these measurements are as follows:

3 $$ \mathrm{DSC}=\frac{2\mathrm{TP}}{\mathrm{FP}+2\mathrm{TP}+\mathrm{FN}} $$

4 $$ \mathrm{sensitivity}=\frac{\mathrm{TP}}{\mathrm{TP}+\mathrm{FN}} $$

5 $$ \mathrm{specificity}=\frac{\mathrm{TN}}{\mathrm{TN}+\mathrm{FP}} $$

The correlation coefficien t[27] between the area calculated from the automatic segmentation results and that calculated from the GT were calculated to evaluate their consistency as follows:

6 $$ \mathrm{R}=\frac{\sigma_{x,y}}{\sigma_x\times {\sigma}_y} $$

## Results

### Local cross-validation results

**Fig. 4:**
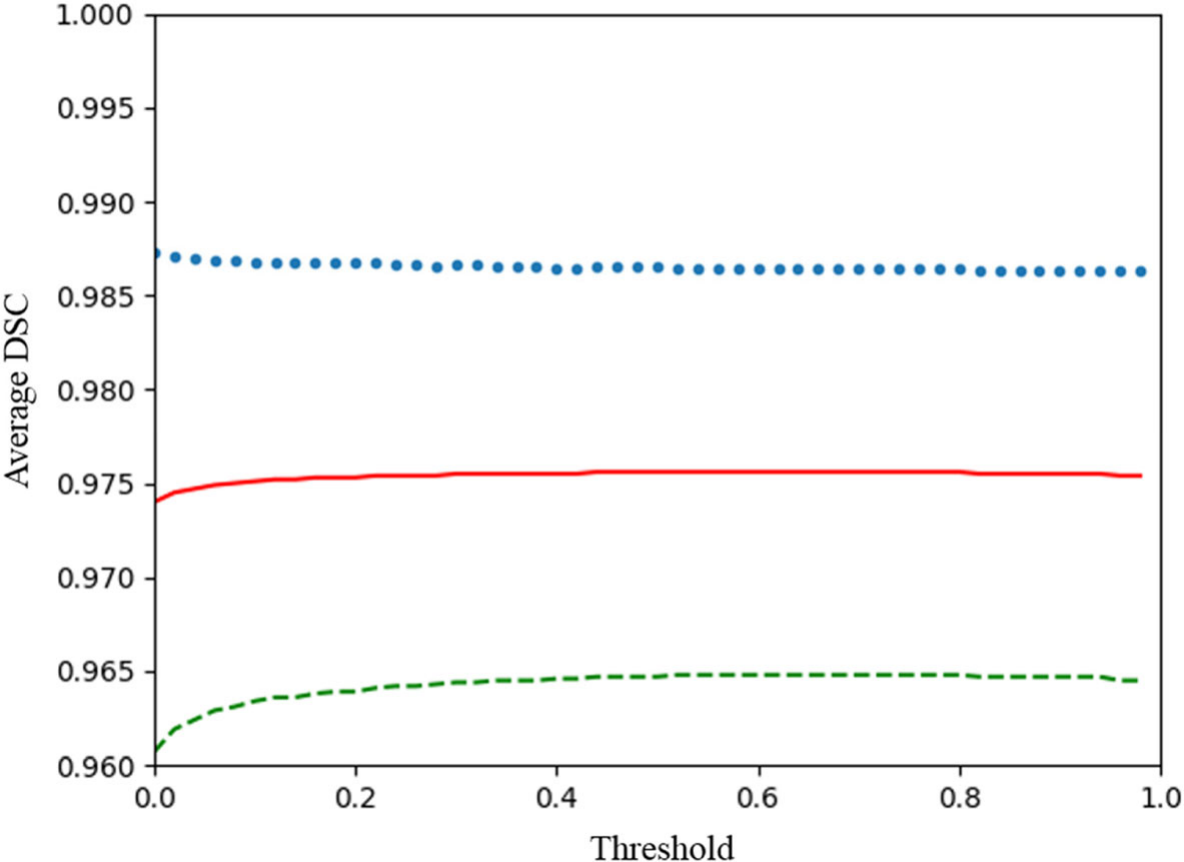
Mean dice similarity coefficient of the proposed method with binarization of different threshold values. The red line denotes the mean DSCs of all deep learning network outputs after binarization of different threshold values; the dashed green line and the dotted blue line denote one standard deviation below and above the mean DSC, respectively. DSC: Dice similarity coefficient

**Fig. 5:**
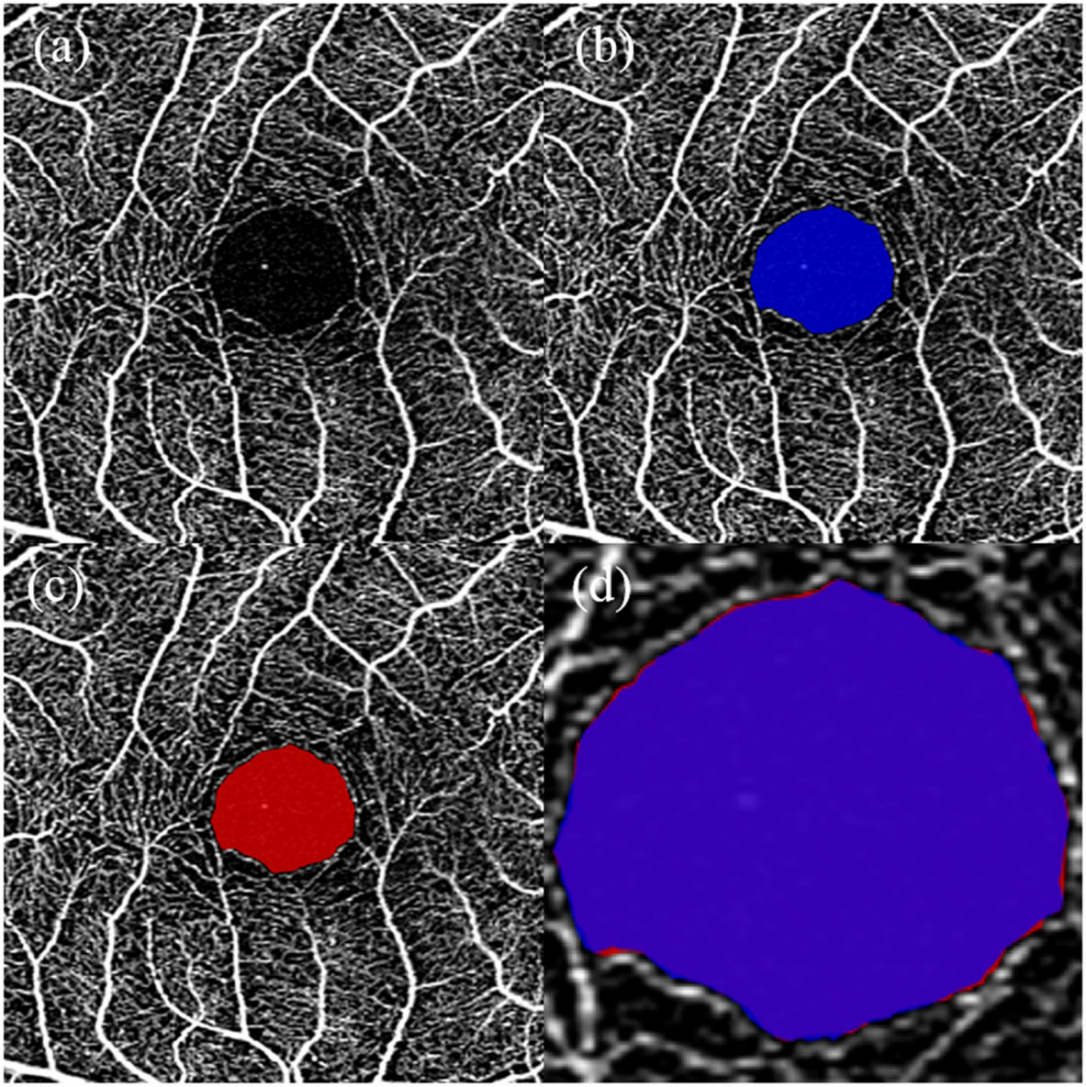
A typical example of automatic superficial foveal avascular zone segmentation. The dice similarity coefficientwas 0.990. **a** Optical coherence tomography angiography (OCTA) image; **b** Automatic segmentation result (blue area) presented on OCTA image; **c** Ground truth (GT) (red area) presented on OCTA image; **d** Magnification of the differences between automatic segmentation results (blue area) and GT (red area); the purple area is the overlapping part of the automatic segmentation results and the GT

The DSC for each OCTA image was calculated and averaged over all OCTA images, as shown in Fig. 4. The red line denotes the mean DSCs of all deep learning network outputs after binarization of different threshold values. The mean DSC increases from the threshold value at 0 with the minimum mean DSC of 0.974 to the threshold value at 0.44 with the maximum mean DSC of 0.976. The mean DSC maintained at 0.976, while the threshold changed from 0.44 to 0.80 and then begins to decline. The maximum mean DSC was obtained in a wide range of thresholds, and the mean DSC variation over the entire threshold range was gradual, which indicates that the performance was insensitive to the selected threshold. A typical example of an automatic sFAZ segmentation with a DSC of 0.990 is shown in Fig. 5. One standard deviation below and above the mean DSCs is marked with a dashed green line and a dotted blue line, respectively. Qualitatively, the deviation of the mean DSC that ranges from 0.011 to 0.013 was small for different thresholds, with the maximum mean DSC of 0.976 ± 0.011 at the threshold value of 0.44, which indicates that the performance was robust and consistent over all OCTA images.

At the threshold value at 0.44, the mean DSC, mean sensitivity, and mean specificity are 0.976 ± 0.011, 0.972 ± 0.019, and 0.999 ± 0.001, respectively, as shown in Table 1. The correlation coefficient between the area calculated from the automatic segmentation results and that calculated from the GT was 0.997, which indicated the significant correlation between the area calculated from the automatic segmentation results and that calculated from the GT.

**Table 1:** Segmentation performance with threshold value of 0.44

	Dice similarity coefficient (mean ± SD)	Sensitivity (mean ± SD)	Specificity (mean ± SD)	R
Current study	0.976 ± 0.011	0.972 ± 0.019	0.999 ± 0.001	0.997 (*p* = 0.000 < 0.5)

We divided all the OCTA image into nine different B/C variation parameter groups according to the description in Section 2.1 and calculated the DSC, sensitivity, specificity, and correlation coefficient in each parameter group based on the threshold value of 0.44, as shown in Table 2. In the nine parameter groups, the mean DSC, mean sensitivity, and mean specificity of the proposed method were higher than 0.96, and the correlation coefficient exceeding 0.99 indicated a significant correlation between the area calculated from the automatic segmentation results and that calculated from the GT.

**Table 2:** Segmentation performance of the proposed method in each parameter group

Parameter group	Dice similarity coefficient (mean ± SD)	Sensitivity (mean ± SD)	Specificity (mean ± SD)	R
G1	0.977 ± 0.001	0.972 ± 0.017	0.999 ± 0.001	0.998 (*p* = 0.000 < 0.5)
G2	0.975 ± 0.011	0.967 ± 0.022	0.999 ± 0.001	0.997 (*p* = 0.000 < 0.5)
G3	0.977 ± 0.001	0.972 ± 0.017	0.999 ± 0.001	0.998 (*p* = 0.000 < 0.5)
G4	0.976 ± 0.010	0.972 ± 0.018	0.999 ± 0.001	0.998 (*p* = 0.000 < 0.5)
G5	0.976 ± 0.010	0.972 ± 0.018	0.999 ± 0.001	0.998 (*p* = 0.000 < 0.5)
G6	0.976 ± 0.010	0.973 ± 0.018	0.999 ± 0.001	0.997 (*p* = 0.000 < 0.5)
G7	0.977 ± 0.009	0.974 ± 0.016	0.999 ± 0.001	0.998 (*p* = 0.000 < 0.5)
G8	0.975 ± 0.011	0.971 ± 0.020	0.999 ± 0.001	0.998 (*p* = 0.000 < 0.5)
G9	0.972 ± 0.013	0.975 ± 0.018	0.999 ± 0.001	0.996 (*p* = 0.000 < 0.5)

### Comparison with other methods

Table 3 shows the comparison of segmentation performance in terms of the DSC and the correlation coefficient between the current results and those of published results using other methods.

**Table 3:** Comparison of the segmentation performance between our proposed method and similar studies

Study	Dice similarity coefficient	R
Lu et al .[12]	0.808	0.792 (*p* = 0.000 < 0.5)
Díaz et al .[13]	0.879	0.666 (*p* = 0.000 < 0.05)
Cheng et al .[28]	0.925	0.948 (*p* = 0.000 < 0.05)
Gharaibeh et al .[29]	0.915	0.940 (*p* = 0.000 < 0.05)
Current study	0.976	0.997 (*p* = 0.000 < 0.5)

## Discussion

In the current study, we propose a segmentation and quantification method based on deep learning for automatically segmenting and quantifying the sFAZ in the OCTA images with the best mean DSC of 0.976. Among the automatic segmentation results of all the OCTA images, the low standard deviation ranging from 0.011 to 0.013 for the mean DSC indicates the robustness of the proposed method. The correlation coefficient between the area calculated from the automatic segmentation results of the proposed method and that calculated from the GT was 0.997, which indicated that the significant correlation was reliable for the automatic quantification of the sFAZ. In the nine different B/C variation groups, the mean DSC of the proposed method was higher than 0.96 and the correlation coefficient was higher than 0.99. This good performance indicates that our method is robust to B/C variations. In the method evaluation, compared to two similar studies in the literature, the proposed method provided the mean DSC of 0.976 and the correlation coefficient of 0.997, which were superior and indicated better accuracy and more significant correlation in sFAZ segmentation. The obtained results also indicated better consistency between the area calculated from the automatic segmentation results and the area calculated from GT with the proposed method than the consistency obtained with other methods, with the best mean DSC and the best correlation coefficient of 0.925 and 0.948, respectively. This might indicate that the proposed method improved the automatic segmentation and quantification accuracy.

Such a good performance may be attributed to the modified U-Net architecture in the current study. The pooling layer in the encoder reduced the size of the feature map and increased the receptive field to efficiently extract representative global information. The decoder was applied to perform size reduction on a small-sized feature map to obtain a prediction image of the same size as the input image. The skip connection structure with an SE block between the encoder and decoder was designed to supplement the decoder with weighted focus information that would provide more sFAZ features as well as compensate for the loss of information during the feature size reduction of the pooling layer.

The second reason for the good performance might be the SE block and BN layer adopted in the current study. The SE block assigned a weight between 0 and 1 for each feature map by feature learning to weigh the sFAZ at the channel level and suppress noise interference. The essence of the deep learning network learning process is to learn the data distribution. To solve the problem of significantly reduced network generalization given the distribution inconsistency of the training data and testing data, we applied normalization to preprocess the data in Section 2.2. However, the distribution of data output at each layer of the network might change as the training progresses. This would not only reduce the network generalization significantly, but also reduce the training speed considerably. Therefore, we applied the BN layer behind the layer (Fig. 2), which might change the original data distribution to achieve a high generalization performance of the network and accelerate the training process. The SE block and the BN layer were utilized to improve the robustness to different B/C versions and noise in the sFAZ.

The proposed deep-learning-based method may exhibit some limitations. The performance of a deep-learning-based method is closely related to the quantity of training data. More data from multicenters should be collected to construct a more robust method and improve the generalization ability of this method.

The dense and complex deep retinal capillary network challenges the observation and quantification of the dFAZ by artificial method s[30] and the extraction of special anatomical information of the dFAZ using program algorithms. A typical challenge of dFAZ segmentation is the low contrast of the dFAZ boundary. The proposed method based on deep learning provided a highly accurate automatic sFAZ segmentation with robustness to B/C variations. This indicates that the proposed method allows an accurate extraction of the anatomical information of the sFAZ and is insensitive to variations in B/C. These demonstrate the great potential of the proposed method for the segmentation of the dFAZ. In future studies, we plan to apply deep learning to the automatic segmentation of the dFAZ.

## Conclusions

In the current study, we proposed and successfully verified an automatic sFAZ segmentation and quantification method based on deep learning with robustness to B/C variations. We improved the U-Net by appending BN layers and SE blocks, which resulted in increased accuracy and generalization. A comparison with the GT indicated that the proposed method demonstrated high accuracy and significant consistency in sFAZ segmentation and quantification with robustness to B/C variations. For clinical analyses, this is a key step in creating an automatic segmentation and quantification of the sFAZ. Future studies will include applying the proposed method to the automatic segmentation of the dFAZ.

## Data Availability

The datasets used and/or analysed during the current study are available from the corresponding author on reasonable request.

## Abbreviations

B/C: Brightness and contrast
BN: Batch normalization
dFAZ: Deep foveal avascular zone
DSC: Dice similarity coefficient
FAZ: Foveal avascular zone
FN: False-negative
FP: False-positive
GT: Ground truth
OCT: Optical coherence tomography
OCTA: Optical coherence tomography angiography
SE: Squeeze-and-excitation
sFAZ: Superficial foveal avascular zone
TN: True-negative
TP: True-positive
